# Chemicals, noise and occupational hearing health in South Africa: A mapping study

**DOI:** 10.4102/sajcd.v67i2.693

**Published:** 2020-03-10

**Authors:** Mershen Pillay

**Affiliations:** 1 Discipline of Speech-Language Pathology, School of Health Sciences, University of KwaZulu-Natal, Durban, South Africa

**Keywords:** Chemical, Ototoxicity, Occupational health, Audiology, Hearing loss, Low- and middle-income countries, Mapping study, South Africa

## Abstract

**Background:**

Chemical exposure leading to ototoxicity is a fresh challenge for occupational healthcare in South Africa.

**Objectives:**

The critical question is: ‘what is known about occupational ototoxic chemicals with or without noise exposure in South Africa?’

**Method:**

This qualitative, mapping study was completed with published (peer-reviewed) and grey literature from 1979-2019. Data was analysed using the Preferred Reporting Items for Systematic reviews and Meta-Analyses: extension for Scoping Reviews and the Nursing and Allied Health Resources Section subcommittee on Mapping the Literature of Nursing and Allied Health (adapted). Numerical analysis of article type was completed, but the primary focus was on capturing patterns/trends using thematic analysis and ideology critique.

**Results:**

The *African Journal of Disability, African Journal of Primary Health Care and Family Medicine, South African Medical Journal, The South African Journal of Communication Disorders* [*SAJCD*] and *Health SA Gesondheid*) were included with the *SAJCD* containing one relevant item and seventeen other items were analysed. Research focusses on the mining sector (gold) in Gauteng, and ototoxic medication (tuberculosis and/or human immunodeficiency virus) take precedence. In KwaZulu-Natal, the focus is on commerce and industry across formal and informal sectors. There are no governmental policies that refer to chemical ototoxicity. Occupational hearing loss is configured exclusively on the meme that noise exposure is the only toxin.

**Conclusion:**

Chemical exposures are only just beginning to be recognised as ototoxic in South Africa. Hearing conservation programmes should always serve the workers’ interests and never bow down to the econometric interests of employers.

## Death and (hearing) disability as collateral damage for workers

Every day, 6000 women and men die because of occupational fatalities. Of those who survive, 160 million live with disabling injuries or illnesses (International Labour Organisation, 2019). Amongst this workforce, hearing loss is the main contributor to disability-adjusted life years for approximately one in five workers (WHO, 2018). This highly preventable disability is a global concern. Occupational noise exposure is a major cause of hearing loss (Basner, 2019). The general perception is that occupational hearing loss is greater in industrialised (minority world) countries like in Australia, New Zealand, North America and other European countries. However, in less industrialised (majority world) regions, ~21% of workers (vs. ~16% in the minority world) experience disabling hearing loss (Basner et al., 2014; Nelson, Nelson, Concha-Barrientos, & Fingerhut, 2005).

In, sub-Saharan Africa, the World Bank recently (2016–2017) commissioned a study on occupational health in the mining sector (Osewe & Nkrumah, 2018). According to this report, hearing loss accounted for 18% of the burden of injury among ex-mine workers in Lesotho, Mozambique, South Africa and Swaziland. Historically, this is not unusual given how migrant workers across the world were (and continue) to work in 3D (dirty, dangerous and difficult) jobs (Pillay, 2013). Osewe and Nkrumah also presented a novel model of worker-centred hearing healthcare across hearing screening, audiological management and aural rehabilitation. Significantly, from an early identification perspective, they stated that during hearing screening (Osewe & Nkrumah, 2018):

If the candidates pass this screening, they would still be eligible for a re-screening every six months to monitor their hearing as part of their being a high-risk group with a history of noise (and/or ototoxic chemical) exposures in the workplace. (p. 173)

This is one of the most significant items in this World Bank’s discussion paper. Why? For hearing healthcare in Southern Africa, the World Bank’s recommendation represents a shift from relying on a ‘noise-only’ discourse to explaining occupational hearing loss. Critically, Osewe and Nkrumah have sounded alarm bells about workplace chemicals as contributing to hearing loss too. This positioning of chemical ototoxicity is a fresh challenge for occupational healthcare in less industrialised world regions such as sub-Saharan Africa.

## Chemicals with or without noise poison peoples’ ears

Among occupational healthcare professionals, it is widely known that chemicals affect hearing. It is well established that medications (chemicals) are ototoxic as many illnesses, and diseases in the world are treated using medications with innate ototoxic properties, for example, aspirin (Johnson & Morata, 2010). Globally, over 600 categories of medications are identified as potentially ototoxic and include aminoglycoside antibiotics, platinum-based chemotherapeutic agents, loop diuretics, macrolide antibiotics and antimalarials (Ganesan, 2018). For example, aminoglycosides (like kanamycin) used to treat tuberculosis (TB) (Khoza-Shangase & Stirk, 2016), highly active anti-retroviral therapy (HAART) for human immunodeficiency virus/acquired immunodeficiency syndrome (Khoza-Shangase, 2009) and cisplatin for cancer (Paken, Govender, Pillay & Sewram, 2016; Whitehorn et al., 2014) are all associated with hearing loss. Notably, these medications also have an impact on the vestibular system, resulting in vertigo or dizziness (Khoza-Shangase, 2018). While there is some appreciation of the ototoxic effects of relevant medications (Wium & Gerber, 2016), chemicals are rarely recognised as ototoxic agents in South African occupational settings and are not identified as contributing to occupational hearing loss (Manning & Pillay, 2020).

Johnson and Morata (2010) reported impact on cochlear and central auditory processing structures owing to ototoxic properties from several solvents, for example, styrene, toluene, xylene, ethylbenzene, trichloroethylene, *n*-hexane, jet fuel, white spirit and other solvent mixtures. Worker-based research studies have focussed mainly on styrene, toluene, lead, mercury, carbon disulphide and carbon monoxide (Johnson & Morata, 2010; Nakhooda, Sartorius, & Govender, 2019). Table 1 shows a review of chemicals (and workplaces) that are potentially ototoxic.

**TABLE 1:** Lists of chemical exposures and workplaces that are potentially ototoxic.

Chemical exposures	Potentially ototoxic workplace
***Solvents***	
Butanol	Aircraft maintenance
Carbon disulphide	Assay labs
Ethanol	Boat building
Ethyl benzene	Construction
*n*-Heptane	Furniture making
*n*-Hexane	Fuelling vehicles and aircraft
Perchloroethylene	Fire fighting
Solvent mixtures and fuels	Painting
Stoddard solvent (white spirits)	Printing
Styrene	Manufacture of metal, fibreglass, leather and petroleum products
Toluene	Pesticide spraying
Trichloroethylene	Radiator repair
Xylenes	Firing of weapons
***Metals*** Arsenic Lead Manganese Mercury Organic tin	
***Others*** Acrylonitrile Carbon monoxide Hydrogen cyanide Organophosphates Paraquat	

Animal experimental studies (Johnson & Morata, 2010) revealed the synergistic effects of noise and solvents (Chen, Chi, Kostyniak, & Henderson, 2007), and demonstrated loss of hair cells hypothesised to be caused by the formation of free radicals, namely, reactive oxygen species (Le Prell et al., 2007). Johnson and Morata (2010) reported other animal studies where chemicals (metals and pesticides) have damaged the cochlea and central auditory pathways. These animal studies are corroborated by studies of workplace chemicals on the human auditory mechanism (see Appendix 1). Ototoxic chemicals may damage the cochlea, vestibular mechanism and/or the auditory neurological pathways, leading to hearing loss, tinnitus and vertigo. The need for evidence regarding noise exposures in combination with workplace chemical exposure has been promoted since the 1990s (Morata & Dunn, 1995). For workers exposed to chemicals and noise, this combined exposure has power to, synergistically, potentiate noise-induced hearing loss (Lewkowski et al., 2019) even when exposures for each agent alone are measured at acceptable levels (Morata, 2007).

In summary, exposure to chemicals in the workplace may be ototoxic. If combined exposures to chemicals and noise occur, then the risk for hearing loss is exacerbated, for example, noise and multiple solvent exposures (Nakhooda et al., 2019) or TB medications use/exposure (Khoza-Shangase, 2018).However, while this is a known global concern, in South Africa, exposure to chemicals in the workplace is barely/hardly documented.

## Why should South African hearing health professionals care?

South Africa’s Apartheid government continued British and other European colonial missions to subjugate black South Africans (Pillay & Kathard, 2018). Mainly white South African employers and international businesses turned a blind eye to the health and safety of their mainly black South African workers (Gumede, 2015). As a South African audiologist having worked under Apartheid and presently working in a post-1994 South Africa, I have observed the continuation of the substandard management of workers’ health. Notably, this occurs under a government born from a coalition with worker unions, a constitution that enshrines worker rights replete with worker-oriented policies, protocols and guidelines.

**TABLE 2:** Included journals with identified data items.

Journal	Included items	Actual period reviewed
African Journal of Disability	0	2012 (first volume) to 2019
African Journal of Primary Health Care & Family Medicine	0	2009 (first volume) to 2019
South African Medical Journal	0	1979–2019
The South African Journal of Communication Disorders [Die Suid-Afrikaanse Tydskrif vir Kommunikasieafwykings]	1	1979–2019
Health SA Gesondheid	0	1996–2019

In Inchanga (KwaZulu-Natal [KZN]) in the early 1990s, I worked with communities maimed by the British company, Thor Chemicals. This company is a case in point of how big business and government, enabled by a historicised geopolitical license, value the dollar over death and disability. In the 1970s, they started exporting toxic chemical waste (mainly mercury) to Cato Ridge (KZN) after disallowed to continue operations in England (Carnie, 2019). So, from Margate in England to Margate in KZN (and beyond), British toxic waste flowed into the Mgweni River and its tributaries, and found its way into the bodies of workers and local residents. Residents 30–40 km away from the city of Durban/eThekwini washed in these rivers, and drank and irrigated food crops with Thor Chemicals’ mercury. Many died, and those residents and workers who survived live as persons with disabilities in contemporary South Africa (Makhaya & Mkhize, 2019) with even more worker exposures documented in October 2019 (Carnie, 2019).

Corporations with social responsibility on their minds and old and new democracies are bedfellows with the ‘unholy trinity’: the International Monetary Fund, World Bank and the World Trade Organisation (Peet, 2009). Big business makes or uses, stores and/or spills chemicals into water, land and air, resulting in death or disability as the outcome for workers. As workplace chemicals are neurotoxic (some predominantly ototoxic), South African audiologists are obliged to get to know not just what these chemicals are but also where/which occupational settings are likely to be considered ‘high-risk’. Such data would inform the management of workers who are vulnerable, key populations at risk of hearing loss, especially when exposed to chemicals with/without noise. Hence, the critical question for this study is: ‘what is known about occupational ototoxic chemicals with/without noise exposure in South Africa?’

## Aim and objectives

The aim of this study was to identify the coverage of the current body of knowledge of workers’ hearing when exposed to ototoxic chemicals with/without noise in literature focussed on South Africa. Topics that have attracted hearing healthcare researchers, and gaps or opportunities for further research will be identified.

## Methodology

The selected design is a qualitative, literature mapping study because it is best suited to answer a research question that is about ‘what is happening’ in a particular subject or field of inquiry. Mapping studies, as a genre of evidence synthesis methods, have been used since the early 1990s by health science researchers. Perryman (2016) reviewed mapping studies relative to other similar methodologies for (1) comprehensiveness, (2) transparency in methodology and (3) rigour. She declared that generic/narrative-type literature reviews are at the one end of a scale, with systematic reviews and meta-analyses on the other end of the scale owing to the nature of their protocols (data retrieval, categorisation, statistical synthesis) and their narrow focus area/topic. Depending on the protocol, mapping studies may – like scoping reviews – also categorise literature through content analysis. Dissimilar to other reviews, mapping studies generally do not involve critical content evaluation or statistical synthesis of findings. Indeed, mapping reviews are often misunderstood as scoping reviews and vice versa. However, instead of analysing results, mapping studies identify the relationship between ideas (Perryman, 2016). Such studies may occur in one of two formats: firstly, they may be formatted vis-à-vis a visualisation of ideas, not unlike mind or concept maps, for example, see an excellent ototoxicity concept map by Watts (2019). Secondly, and not mutually exclusive, studies are characterised by varying levels of systematic mapping of, for example, where an activity occurred, research funding sources, journal site or mode/medium of presentation. This second format, selected for my study, may include either published, peer-reviewed literature or other media like books, newspapers, policy documents and Internet/electronic data (Cooper, 2016).

## Data sources

Two broad data sets were used: (1) published, peer-reviewed articles – obtained via two search strategies, detailed below, and (2) grey literature (including print/paper data) tagged by the type or authenticity of the data, see below. Databases used were limited to Google Scholar, PubMed/Medline and ScienceDirect/Scopus for a 40-year period (1979–2019) – selected for accessibility of electronic and print data.

## Sampling

Theoretical or operational construct sampling was used because this form of purposive sampling is suited for the selection of data that represent important constructs about the phenomenon of interest (Suri, 2011). Operational definitions were developed for the following constructs: chemical ototoxicity with/without noise exposures and occupational hearing loss. A data richness scale was developed (simplified after Ames, Glenton, & Lewin, 2019) to rate the amount of data (as per analytical categories, detailed below) in the selected item (e.g. ‘1’ = little/fair data, ‘5’ = large/rich data), and a strategy of sampling for (match of) study scope (cf. inclusion criteria) was used to guide data items selected for mapping.

## Search strategy

As the design is a mapping study but with principles adapted from other evidence synthesis methods like scoping and systematic literature reviews, two search strategies were used; the initial search (discussed in detail below) was performed by a research assistant. Firstly, we identified highly ranked journals via the international Scopus^1^ *SCImago Journal* and Country Rank that were likely to publish articles regarding occupational hearing loss owing to chemical ototoxicity with/without noise, focussed on South Africa. There are inherent limitations to this method, discussed later. However, words (or synonyms) in the journal titles had to be related to ‘occupation’ and/or ‘health’, ‘hearing’, ‘disability’, ‘communication’, ‘noise’, ‘pharmaceutical’, ‘chemical’ and/or ‘toxicology’. ‘African’ and/or ‘South(ern) Africa’ and synonyms were the terms selected to identify and include relevant journals.

Secondly, a search strategy was adapted from the Preferred Reporting Items for Systematic Reviews and Meta-Analyses Extension for Scoping Reviews (PRISMA-ScR) (Tricco et al., 2018). Within this strategy, and given that the Scopus (2018) *SCImago Journal* and Country Rank is limiting by geopolitical intellectual space, I was concerned about a bias/risk of missing unindexed publications from/about South Africa. This then made it necessary to locate articles that were published but not necessarily peer-reviewed, namely, grey literature like items in paper (vs. electronic) versions, for example, unpublished university student dissertations. Across peer-reviewed published and grey literature items, I ensured that all were about South African workers, chemical (with/without) noise exposures and occupational hearing loss. Therefore, keywords searches were completed using medical subject headings (MeSH) or Boolean searches (as per database) for ‘chemicals’, ‘solvents’, ‘hearing loss’, ‘ototoxicity’, ‘audiologist’, ‘audiology’, ‘hearing healthcare’, ‘South Africa’, ‘Africa’, ‘industry’ and ‘occupational health and safety’.

## Inclusion and exclusion criteria for articles

To develop boundaries, specific inclusion and exclusion criteria were created to facilitate the selection of primary studies for synthesis. The inclusion criteria were that the article content had to focus on: (1) chemical exposures with/without noise in the workplace and that (2) the South African workforce were considered for the time period stipulated above (1979–2019).

## Data collection and analysis

A structured data collection schedule was developed and included the following items:

1. A determination of the number of citations and classification of the literature format types by:
  - books
  - government documents
  - Internet source
  - miscellaneous.
2. Database sorting of format type, cited year (selected as 1979 onwards) and tagged as:
  - ‘pre-’ if prior to the year range
  - ‘in-press’ for unpublished work in 2019
  - ‘unknown’ for undated items.
3. When journals changed their titles/names, articles were listed under the most recent title of the journal.

*Development of the data collection tool and process*: A data collection schedule was adapted from the protocols of the Nursing and Allied Health Resources Section (NAHRS) Subcommittee on Mapping the Literature of Nursing and Allied Health (Delwiche, Schloman, & Allen, 2010). Nursing and Allied Health Resources Section protocols are premised on Bradford’s Law of Scattering (Cooper, 2016). This law prescribes reviewing journals over 3 years (not followed here) to establish bibliographic coverage and zoning as to how ‘scattered’ the topic is across specific journals. This has the potential to influence database producers because it characterises and zones data by accessibility. Therefore, in this study, Bradford’s Law of Scattering is applied in principle towards establishing/zoning coverage of the knowledge scatter and not towards quantitative calculations.

Initially, a research assistant and I completed the selection of whole journals by title and specific journal articles were selected by title and abstract. This was a blind review in an attempt to increase inter-rater consistency. We resolved conflicts via discussion and in defence of our application of the inclusion/exclusion criteria, removed duplicates and finalised a single list of journals and articles to review. Notably, where duplicates occurred between published and grey literature, for example, Brits (2011) (an unpublished master’s thesis) and Brits, Strauss, Eloff, Becker and Swanepoel (2012) (a published, peer-reviewed item), the latter was selected as superior. The same principle applied within grey literature items like, for example, Edwards (2009) (a conference paper) and Edwards (2012) (a conference poster), the former was selected over the poster version. Once the initial search was completed, I conducted all further article searches (by title and abstracts) in each journal, searched for grey literature and finally conducted an analysis of all selected data items.

*Analysis and interpretation of data:* The emergent-focussed mapping review and synthesis method (Aveyard & Bradbury-Jones, 2019) was considered to examine broader epistemological contexts of the knowledge scatter in chemical ototoxicity.

Notably, titles and abstracts were also analysed in this adaption of the PRISMA-ScR as per guidelines by Schultz et al. (2018). Therefore, all selected items were read in full and data were extracted as per the data analysis schedule, adapted from NAHRS (described above). This implies that abstracts constituted full data items and were not up for exclusion but analysed for their content. Numerical analysis of the number of data items by type was computed. The primary focus for this analysis was on capturing patterns and trends in the literature. To do this, data were analysed using Braun and Clarke’s (2006) method beginning with: (1) data familiarisation; (2) initial coding; (3) theme identification; (4) theme clustering; (5) theme definition towards a conceptual topography/map; (6) defining/naming themes towards overall patterns; and (7) report of empirical data for analysis. I also performed an ideological critique based on an interrogative framework by Guba and Lincoln (1994), adapted by Pillay (2003) and Pillay and Kathard (2018), and supported with exemplary textual extracts from included articles to demonstrate claims made. No computer software was used in the analysis of the data. Neither were any critical quality appraisals of methodology completed as the purpose was to profile what is happening in the field rather than to draw conclusions from the included studies’ findings.

Several methods were used during the data collection and analysis phases to ensure credible, trustworthy data such as establishing the authenticity of data sources via ranked journals. Triangulation of multiple data sources and multiple data collection methods was used to increase trustworthiness of the overall study. In this way, triangulation assisted in a comprehensive understanding of chemicals and hearing loss in the workplace by ‘testing’ validity through data convergence from different sources (grey, published). The initial blind review for the selection of primary data assisted with data credibility too. Data dependability was checked via a detailed track record of the data collection process. As generalisability is not the goal of this type of qualitative research, transferability was what guided evaluations of the usefulness of data for other contexts or setting conducted via designing reflexive categories of analysis.

### Ethical considerations

This research received no specific grant from any funding agency in the public, commercial or not-for-profit sectors.

## Results and discussion

In relation to stated aims and objectives, South African workers’ exposure to chemicals with/without noise is discussed under the following topics:

1. current knowledge
2. focal research topics
3. knowledge gaps and research opportunities.

## Current knowledge

The following journals were identified using the International Scopus (2018) *SCImago Journal* and Country Rank for South Africa only, and by the following subject areas: ‘Health Professions’, ‘Medicine’, Multidisciplinary’, ‘Neuroscience’ and ‘Pharmacology, Toxicology and Pharmaceutics’:

1. African Journal of Disability
2. African Journal of Primary Health Care and Family Medicine
3. South African Medical Journal
4. The South African Journal of Communication Disorders [Die Suid-Afrikaanse Tydskrif vir Kommunikasieafwykings]
5. Health SA Gesondheid

Journal contents’ pages were reviewed (40-year period as per electronic and print media accessibility) for a focus on chemical exposures with/without noise regarding South African workers. Notably, there were no occupational health-related journals ranked by the *SCImago Journal* and Country Rank, in spite of the *Occupational Health Southern Africa* journal being accredited by the Department of Higher Education and Training in South Africa. Across all the journals, only one item per journal was included, which was the same as that identified in the data base searches (Nakhooda et al., 2019).

**FIGURE 1:**
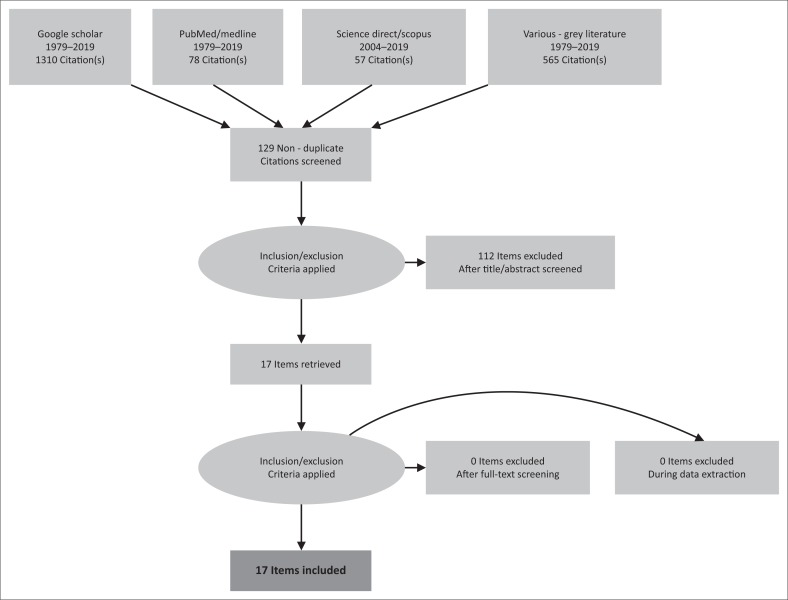
Preferred Reporting Items for Systematic Reviews and Meta-Analyses Extension for Scoping Reviews flow chart for the mapping review of knowledge about workers’ hearing when exposed to ototoxic chemicals with/without noise in literature focussed on South Africa.

Preferred Reporting Items for Systematic Reviews and Meta-Analyses Extension for Scoping Reviews guidelines were used to electronically identify the initial data set of 2010 items. Inclusion criteria were applied – see the PRISMA-ScR flow chart (Figure 1), adapted from the PRISMA scoping review guidelines for this mapping study.

Table 3 contains the final included set of 17 items classified by NAHRS categories. Besides six published articles, items included in the data set consist of grey literature items, namely, a professional association (SASLHA) conference programme, a conference paper (full, unpublished) and abstracts of conference presentations. A global discussion paper, published by the World Bank with expert peer input but not peer reviewed, was also included.

**TABLE 3:** Data items classified by type.

Type of sources	Total number (17)
**Internet sources**	
Master’s thesis	1
Doctoral/PhD thesis	2
Published paper	6
Conference paper (full)	1
Conference paper (abstract)	3
Conference poster (abstract)	1
Abstract (paper)	1
**Miscellaneous**	
Conference Programme (print)	1
Books	0
Global discussion paper	1
Government documents	0

Table 4 shows an overview of each item including an evaluation of data richness and sample matching as per inclusion criteria. Notably, three items (Nakhooda, 2016; Nakhooda et al., 2019; Niranjan 2015) are the most suitable for analysis. It may be noted that authors, where possible, were identified as mainly audiologists and/or from community health studies (Niranjan). Most study sites referred to the mining sector, specifically gold mines in Gauteng and environs. Other sites, when stated, included the paint and shoe industries, rubber industries and the informal sector, namely, street market traders that encompassed both urban environmental exposures and other toxins.

**TABLE 4:** Item classification by type, data richness and sampling match.

Item	Data type	Data richness rating	Sampling match
Brits et al. (2012)	Published paper	4	3
Edwards and Franz (2009)	Published paper	4	3
Edwards (2009)	Conference paper (full)	4	5
Edwards (2013)	Abstract (paper)	3	5
Khoza-Shangase (2018)	Published paper	5	4
Moroe, Khoza-Shangase, Madahana, and Nyandoro (2018)	Published paper	5	3
Nakhooda (2016)	Master’s thesis (study)	5	5
Nakhooda et al. (2019)	Published paper	5	5
Niranjan (2014)	Doctoral thesis	5	5
Osewe and Nkrumah (2018)	Global discussion paper	4	4
Pillay (2002)	Conference poster (abstract)	3	4
Pillay (2006)	Conference programme (print)	3	5
Pillay (2012)	Conference paper (abstract)	4	4
Pillay and Kinnear (2014)	Conference paper (abstract)	4	4
Pillay and Booi (2018)	Conference paper (abstract)	4	5
Pillay and Kathard (2018)	Published paper	4	2
Strauss (2012)	D. Phil thesis	4	3

## Focal research topics

Most items were focussed on the topic of noise as the point of entry towards understanding chemical ototoxicity in the workplace. All items explicitly refer to combined exposures to chemicals in the workplace. Some make minor references to chemical exposures in their theoretical framework like Edwards (2009). She theorised a ‘…synergistic effect of chemicals and noise on the inner ear’ (p. 2) in her study focussed on multiple risk factors within a synergistic framework, namely, noise, heat and exercise. Significantly, Brits et al. (2012) and Khoza-Shangase (2018) both researched medication-induced ototoxicity (TB) and its ability to potentiate hearing loss by noise in gold mines. Nakhooda (2016) and Nakhooda et al. (2019) are two items emanating from the same study with the latter a systematic review and meta-analysis of the literature. While generated in South Africa and a precursor to the main study (Nakhooda, 2016), this literature review decontextualised South African occupational contexts in favour of analysing combined exposures (noise and solvents) as a phenomenon across global literature bases. Niranjan (2015) completed a study in 2014 on combined exposures in rubber factories that produced components for motor, shoe and plumbing industries in the metropolitan area of Durban. Nakhooda’s main study overlaps with Niranjan’s study as she focussed on workers in the paint and shoe industry. These are the only two South African studies that focussed on noise-chemical exposures and both are based in KZN. My ongoing practice/clinical service project (called *AWEH* – Advancing Workers’ Engagement with Hearing) (see item by Pillay & Kathard, 2018) is also based in KZN. This service, alongside the research generated from/about occupational hearing loss, places KZN as the province that promotes audiologists, hearing healthcare of workers and research in occupational ototoxicity. My coauthors (Booi, Kinnear and Kathard) and I have presented conference papers, posters and a publication, with a specific angle on chemical occupational ototoxicity. This work initially focussed on the rubber industry (Pillay, 2002), with a current focus on workers in the informal sector, namely, street market traders and the complexity of exposures to car fumes, solvents, related ototoxic medications, smoking, alcohol and music and noise exposures in eThekwini’s Warwick Junction markets (see items 11–16).

In summary, research in chemicals and noise exposures in the workplace focus on the mining sector in Gauteng with the interaction of workers on ototoxic medication (for TB and/or HIV) taking precedence. In KZN there appears to be a clearer focus on commerce and industry – both in the formal and informal sectors. KwaZulu-Natal research and services are developed especially for workers exposed to solvents in combination with noise and other risk factors like ototoxic medication.

## Knowledge gaps and research opportunities

The most critical gap noted is that there are no governmental policies that link chemical ototoxicity in any South African policy on occupational hearing loss, noise or otherwise. Occupational hearing loss is configured exclusively on the meme that noise exposure is the only toxin. For a more detailed analysis of this bias and/or omission in the law, see Manning and Pillay (2020).

Furthermore, hearing healthcare professional education programmes must consider the informal sector and workers’ exposure to a plethora of toxins. The notion of occupational hearing loss owing to ototoxicity must find its way onto the agendas of public and occupational health researchers and practitioners alike. Chemical exposures are only just being recognised as ototoxic in the mining (Osewe & Nkrumah, 2018) and other commercial sectors, but this recognition is missing for how workers in the informal sector are managed. While not even noise is managed in the informal occupational sector as yet (Pillay & Kathard, 2018), South Africa’s recognition of chemical ototoxicity in the occupational sector must include informal workers too as we teach, research and develop policies in occupational hearing healthcare.

## Conclusion

In response to the question, ‘what is known about occupational ototoxic chemicals with/without noise exposure in South Africa?’, this study has mapped the knowledge we are developing in this field in South Africa. However, it does indicate the need for a more systematic evaluation of the nature of studies, their methodological quality and a deeper evaluation of the contribution hearing healthcare researchers make in transforming how employers configure their employers’ healthcare.

In a study, entitled ‘Hearing Conservation Programmes: For Employers/Workers?’, South African formulae for calculating workers’ compensation were evaluated for how one accounted for complexities like chemical ototoxic occupational hearing loss (Pillay, 2002). What was critiqued almost 20 years ago remains true even today: South African businesses, like mining companies, maintain a strong interest in commercial gain over healthcare. Notions like corporate social responsibility lie uncomfortably when placed next to workers who are econometric instruments in a business. As healthcare professionals, we need to recognise the inherent conflict with hearing conservation activities when considering >85 dB and solvents; African language speakers and speech audiometry – or the measurement of occupational hearing impairment versus hearing disability. Our hearing conservation programmes, especially when confounded by chemical exposures, noise and other factors, should always serve the workers’ interests and never bow down to the econometric interests of employers.

## Footnotes

1 See https://www.scimagojr.com/.

## Appendix 1: Ototoxic medications by type of effect (hearing loss, tinnitus, vertigo)

The tables below document ototoxic medications by type of effect (hearing loss, tinnitus and vertigo).

**TABLE 1-A1:** Drugs causing hearing loss as a side effect.

Drug category	Class of drug	Subclass of drug	Examples
Anti-infective	Antibiotics	Aminoglycosides	Neomycin, gentamicin, amikacin, netilmicin
	-	Macrolides	Erythromycin, azithromycin, clarithromycin
	-	Quinolones	Ofloxacin, ciprofloxacin, levofloxacin
	-	Others	Tetracyclines, vancomycin, teicioplanin, framycetin, colistin, imipenem with cilastin
	Antivirals	-	Ganciclovir, zalcitabine, ribavirin + interferon
	Antifungals	-	Amphotericin, Flucytosine
	Antimalarials	-	Chloroquine, mefloquine, quinine
	Antituberculous	-	Capreomycin
Analgesics	Non-steroidal	-	Aspirin, indomethacin, ibuprofen, diclofenac
	Anti-inflammatory	-	Ketorolac, sulindac, naproxen
Anticancer drugs	Cytotoxics	Platinum compounds	Cisplatin, carboplatin, oxaloplatin
	-	Vinca alkaloids	Vindesine, vinblastine, vincristine
	-	Others	Bexarotene, taxane
Cardiac and vascular drugs	Diuretics	Loop diuretics	Frusemide, bumentanide, torasemide
	-	Carbonic anhydrase inhibitor	Acetazolamide
	Beta blockers	-	Metoprolol, sotalol, practolol, bisopralol
	ACE inhibitor	-	Ramipril
Neurologic drugs	Anticonvulsant	-	Sodium valproate
	Anti-Parkinson’s	-	Entacapone

*Source*: Bisht, M., & Bist, S.S. (2011). Ototoxicity: The hidden menace. *Indian Journal of Otolaryngology, Head and Neck Surgery, 63*(3), 255–259. https://doi.org/10.1007/s12070-011-0151-8

**TABLE 2-A1:** Drugs causing tinnitus as a side effect.

Drug category	Class of drug	Subclass of drug	Examples
Anti-infective	Antibiotics	Aminoglycosides	Tobramycin, netilmicin, amikacin
	-	Macrolides	Clarithromycin, azithromycin
	-	Quinolones	Ciprofloxacin, ofloxacin, norfloxacin
	-	Others	Tetracyclines, vancomycin, teicioplanin, cotrimoxazole, cefpodoxime, linezolid
	Antivirals	-	Ganciclovir, zalcitabine, Amphotericin
	Antifungals	-	Mefloquine, quinine
	Antimalarials	-	Capreomycin
	Antituberculous	-	-
Analgesics diclofenac	Non-steroidal	-	Aspirin, indomethacin, ibuprofen, diclofenac
	Anti-inflammatory	-	Ketorolac, sulindac, naproxen, celecoxib
Anticancer drugs	Cytotoxics	Platinum compounds	Cisplatin, carboplatin
	-	Vinca alkaloids	Vindesine
	-	Others	Bexarotene, paclitaxel
Cardiac and vascular drugs	Diuretics	Loop diuretics	Frusemide, torasemide
	-	Potassium sparing diuretic	Amiloride
	-	Carbonic anhydrase inhibitor	Acetazolamide
	Beta blockers	-	Metoprolol, timolol
	Alpha blockers	-	Prazocin
	ACE inhibitor	-	Ramipril, Enalapril, trandolapril
	AT-II receptor	-	Irbesartan
	Antagonist	-	-
	Anti-arrhythmic	-	Flecainide, quinidine, adenosine
	Calcium channel blockers	-	Diltiazem, nicardepine
Neurologic drugs	Anticonvulsant	-	Carbamazepine, fosphenytoin
	Anti-depressant	Tricyclics	Imipramine, amitriptyline
	-	SSRI	Citalopram
	Antimigraine	5HT-1 antagonist	Almotriptan
	Hypnotics	Benzodiazepine	(on withdrawal)
Others	Immunosuppressant	-	Tacrolimus
	Antirheumatoid	-	Hydroxychloroquine
	Local anaesthetics	-	Lignocaine
	Hypoglycaemics	-	Tolbutamide
	Antihistamines	-	Chlorpheniramine

*Source*: Bisht, M., & Bist, S.S. (2011). Ototoxicity: The hidden menace. *Indian Journal of Otolaryngology, Head and Neck Surgery, 63*(3), 255–259. https://doi.org/10.1007/s12070-011-0151-8

**TABLE 3-A1:** Drugs causing vertigo or dizziness as a side effect.

Drug category	Class of drug	Subclass of drug	Examples
Anti-infective	Antibiotics	Aminoglycosides	Tobramycin, gentamicin, amikacin, netilmicin
	-	Macrolides	Erythromycin, azithromycin, clarithromycin
	-	Quinolones	Ofloxacin, ciprofloxacin, levofloxacin, norflox
	-	Penicillins	Piperacillin, amoxicillin
	-	Cephalosporins	Cefpodoxime, cefadroxil, ceftazidime, cefixime, cefalexin, cefaclor, cefazolin, ceftriaxone, cephradine
	-	Others	Tetracyclines, vancomycin, teicioplanin, metronidazole, tinidazole, clindamycin, cotrimaxazole, linezolid, pentamidine
	Antivirals	-	Ganciclovir, zalcitabine, acyclovir, zidovudine, amantadine, ritonavir, lopinavir, indinavir
	Antifungals	-	Fluconazole, flucytosine, itraconazole, terbinafine, gresiofulvin
	Antimalarials	-	Hydroxychloroquine, mefloquine, lumefantine
	Antituberculous	-	Isoniazid, rifampicin, capreomycin, cycloserine
	Antihelminthics	-	Piperazine
Analgesics	Non-steroidal	-	Aspirin, indomethacin, ibuprofen, diclofenac
	Anti-inflammatory	-	Ketorolac, sulindac, naproxen, celecoxib, mefenamic acid, aceclofenac, rofecoxib
	Opioids	-	Morphine, codeine, alfentamil, pethidine, tramadol, dextropropoxyphene
Anticancer drugs	Cytotoxics	Platinum compounds	Cisplatin
	-	Vinca alkaloids	Vinblastine
	-	Antimetabolites	Capecitabine, methotrexate, cytarabine
	-	Others	Etoposide, hydroxyurea, procarbazine
Cardiac and vascular drugs	Diuretics	Loop diuretics	Frusemide, bumentanide, torasemide
	-	Thiazides	Indapamide, metolazone, bendrofluazide
	-	Carbonic anhydrase inhibitor	Acetazolamide, dorzolamide
	-	Potassium sparing	Amiloride, spironolactone
	Beta blockers	-	Metoprolol, timolol, propranolol, atenolol, pindolol, sotalol, labetolol, carvedilol
	Alpha blockers	-	Prazocin, terazosin, doxazocin, tamsulosin
	ACE inhibitor	-	Ramipril, Enalapril, trandolapril, captopril, perindopril, lisinopril
	AT-II receptor antagonist	-	Irbesartan, losartan, candesartan, valsartan
	Anti-arrhythmic	-	Flecainide, quinidine, adenosine, digoxin, amiodarone, bretylium, disopyramide
	Calcium channel blockers	-	Amlodipine, nifedipine, verapamil
	Nitrates	-	Isosorbide mononitrate, glyceryl trinitrate
Neurologic drugs	Anticonvulsant	-	Sodium valproate, carbamazepine, fosphenytoin, gabapentin, tiagabine, lamotrigine, ethosuccimide
	Anti-depressant	Tricyclics	Imipramine, amitriptyline, amoxapine
	-	SSRI	Citalopram, fluoxetine, sertraline
	-	MAO inhibitors	Moclobemide
	Antimigraine	5HT-1 antagonist	Almotriptan, sumatriptan
	Hypnotics	Benzodiazepine	Clonazepam, lorazepam, diazepam, midazolam, alprazolam
	-	-	Zopiclone, zolpidem
	Anti-Parkinson’s	Other hypnotics	Entacapone, selegiline, biperiden, bromocriptine, pramipexole
	Antipsychotics	-	Thioridazine, olanzapine, clozapine, chlorpromazine, haloperidol, serindole
	Drugs for dementia	-	Memantine, galantamine, donepezil
	Muscle relaxant	-	Dantrolene, baclofen, tizanidine
Endocrine and metabolic	Hyperglycemics	-	Glipizide, glimepiride, pioglitazone, insulin
	Corticosteroids	-	Dexamethasone, fludrocortisone
	Bisphosphonates	-	Pamidronate, zoledronate
Gastro intestinal drugs	Antiemetics	-	Metoclopramide, ondansetron, nabilone, trifluoperazine, tropisetron
	Antiulcer	H2 antagonist	Ranitidine, cimetidine, famotidine
	-	Proton pump	Omeprazole, lasanoprazole, pantaprazole
	-	Inhibitors	Promethazine, cetrizine, cyclizine
	Lipid regulating	-	Fenofibrate, simvastatin, colestipol
Others	Immunosuppressant	-	Tacrolimus, azathioprine, mycophenolate
	Antirheumatoid	-	Leflunomide, etanercept, azathioprine
	Anti-gout	-	Allopurinol
	Local anaesthetics	-	Ropivacaine, lignocaine
	Antihistamines	-	Chlorpheniramine, fexofenadine, promethazine, cetrizine, cyclizine
	Antimuscarinicss	-	Atropine, hyoscine, dicyclomine
	Antiasthamatics	-	Salbutamol, montelukast, salmeterol

*Source*: Bisht, M., & Bist, S.S. (2011). Ototoxicity: The hidden menace. *Indian Journal of Otolaryngology, Head and Neck Surgery, 63*(3), 255–259. https://doi.org/10.1007/s12070-011-0151-8
